# Effect of emodin on long non‐coding RNA‐mRNA networks in rats with severe acute pancreatitis‐induced acute lung injury

**DOI:** 10.1111/jcmm.15525

**Published:** 2021-01-12

**Authors:** Caiming Xu, Yalan Luo, Michael Ntim, Weili Quan, Zhaoxia Li, Qiushi Xu, Liu Jiang, Jingwen Zhang, Dong Shang, Lei Li, Guixin Zhang, Hailong Chen

**Affiliations:** ^1^ Department of General Surgery The First Affiliated Hospital of Dalian Medical University Dalian China; ^2^ Institute (College) of Integrative Medicine Dalian Medical University Dalian China; ^3^ Department of Physiology Dalian Medical University Dalian China; ^4^ Center for Genome Analysis ABLife Inc Wuhan China; ^5^ Endoscopy Center The First Affiliated Hospital of Dalian Medical University Dalian China; ^6^ Department of Vascular Surgery The Second Affiliated Hospital of Dalian Medical University Dalian China

**Keywords:** acute lung injury, acute pancreatitis, emodin, lncRNA, mRNA

## Abstract

Long non‐coding RNAs (lncRNAs) contribute to disease pathogenesis and drug treatment effects. Both emodin and dexamethasone (DEX) have been used for treating severe acute pancreatitis‐associated acute lung injury (SAP‐ALI). However, lncRNA regulation networks related to SAP‐ALI pathogenesis and drug treatment are unreported. In this study, lncRNAs and mRNAs in the lung tissue of SAP‐ALI and control rats, with or without drug treatment (emodin or DEX), were assessed by RNA sequencing. Results showed both emodin and DEX were therapeutic for SAP‐ALI and that mRNA and lncRNA levels differed between untreated and treated SAP‐ALI rats. Gene expression profile relationships for emodin‐treated and control rats were higher than DEX‐treated and ‐untreated animals. By comparison of control and SAP‐ALI animals, more up‐regulated than down‐regulated mRNAs and lncRNAs were observed with emodin treatment. For DEX treatment, more down‐regulated than up‐regulated mRNAs and lncRNAs were observed. Functional analysis demonstrated both up‐regulated mRNA and co‐expressed genes with up‐regulated lncRNAs were enriched in inflammatory and immune response pathways. Further, emodin‐associated lncRNAs and mRNAs co‐expressed modules were different from those associated with DEX. Quantitative polymerase chain reaction demonstrates selected lncRNA and mRNA co‐expressed modules were different in the lung tissue of emodin‐ and DEX‐treated rats. Also, emodin had different effects compared with DEX on co‐expression network of lncRNAs Rn60_7_1164.1 and AABR07062477.2 for the blue lncRNA module and Nrp1 for the green mRNA module. In conclusion, this study provides evidence that emodin may be a suitable alternative or complementary medicine for treating SAP‐ALI.

## 1 INTRODUCTION

Acute pancreatitis (AP) is an inflammatory disease of the pancreas, diagnosed by abdominal pain and increased concentrations of serum amylase and lipase.^1^ In 80% of AP patients, the pancreatic injury is mild and recovery is without complication.^1^ However, 15%‐20% of AP patients will develop severe acute pancreatitis (SAP) and this can have an overwhelming mortality rate of 20%‐30%.^2^ Acute lung injury (ALI) and acute respiratory distress syndrome (ARDS) are the most common and early remote organ complications of SAP, which can result in a poor prognosis. ALI has been reported to occur in approximately 10%‐25% of all AP cases, accounting for 60%‐70% of SAP‐associated deaths within the first seven days.^3^, ^4^ However, the mechanisms involved in SAP‐ALI remain unclear, and effective drug treatments for SAP‐ALI are limited.^5^ Therefore, it is necessary to explore the mechanisms and potential treatment strategies for SAP‐ALI.

Many animal models have been established to investigate SAP‐ALI pathogenesis as well as to assess potential drug effectiveness.^6^ The therapeutic effects of dexamethasone (DEX) and other drugs have been evaluated in these animal models and SAP‐ALI patients.^7^, ^8^, ^9^, ^10^ It is notable that DEX decreases inflammatory mediators but fails to reduce tissue injury in the lungs of SAP rat models.^10^ Emodin is a natural anthraquinone derivative, which is isolated from the roots and rhizomes of numerous plants, moulds and lichens.^11^ Emodin is an active ingredient derived from the Chinese herbal medicine known as rhubarb, which has multiple therapeutic and pharmacological activities including but not limited to anti‐inflammation, immunosuppression, anti‐fibrosis and anti‐neoplastic activities.^12^, ^13^, ^14^, ^15^ Previous studies demonstrated that emodin significantly decreased mortality in SAP rats and produced therapeutic effects by reducing intestinal mucous, maintaining an optimal intestinal flora, enhancing peristalsis of the intestines, elevating secretory immunoglobulin A levels, inhibiting caspase‐1 and NF‐κB activation as well as downstream inflammatory cytokine release, inducing circulating neutrophil apoptosis and scavenging oxygen free radicals.^16^, ^17^, ^18^, ^19^, ^20^, ^21^, ^22^ Our previous research demonstrated than emodin up‐regulates aquaporin‐5 and aquaporin‐1 expression as well as reduces claudin‐4 and claudin‐5, which contribute to the prevention of pulmonary oedema and protect the alveolar epithelial barrier in SAP‐ALI rats.^23^, ^24^ Thus, emodin may target diverse signal transduction cascades, which in combination with other drugs may be a useful treatment for inflammatory diseases.^11^ However, more work is required to fully evaluate the therapeutic effect of DEX and emodin, as well as their potential molecular mechanisms of action.

Multiple studies have demonstrated non‐coding RNAs (microRNAs, lncRNAs and circRNAs) to interact with each other and to target human genes. These RNAs play important roles in the pathogenesis of diseases and are therefore potential biomarkers and therapeutic targets for these diseases.^25^, ^26^, ^27^, ^28^ In fact, miR‐21 is overexpressed in a murine model of acute pancreatitis, affecting RIP3‐dependent pathologic necrosis.^29^ Abnormal levels of plasma microRNAs may be potential biomarkers that can predict ALI after SAP.^30^, ^31^ Moreover, miR‐542‐5p and miR‐339‐3p protect mice from ALI and SAP by different cell signalling pathways.^5^, ^32^ Further, emodin alleviates sodium taurocholate‐induced pancreatic acinar cell injury via microRNA‐30a‐5p‐mediated inhibition of inflammatory signalling pathways.^33^ Furthermore, emodin attenuates apoptosis and inflammation induced by LPS through the up‐regulation of the lncRNA, TUG1, in murine chondrogenic ATDC5 cells.^34^ It is notable that lncRNAs regulate transcription and influence mRNA processing and post‐transcriptional regulation.^35^, ^36^ Hence, emodin may function through lncRNA‐mRNA networks. However, to the best of our knowledge, there is no evidence of the effect of emodin on lncRNA‐mediated signal transduction networks in SAP‐ALI.

In this study, we have been suggested that the therapeutic effects of emodin and DEX in SAP‐ALI rats are associated with different lncRNAs‐mRNA co‐expression networks. To validate this hypothesis, transcriptome analysis of lung tissues was performed to assess whether lncRNAs‐mRNAs co‐expression networks are associated with the pathogenesis and drug treatment outcomes of SAP‐ALI rats. The purpose of this study is to identify differences in the therapeutic effects of emodin and DEX in rats with SAP‐ALI. Particularly, we assessed whether emodin and DEX ameliorate SAP‐ALI outcomes by different lncRNA‐mediated signal transduction networks in rats. The results of this study will advance the current knowledge and provide evidence to support the use of emodin as a suitable alternative or complementary medicine to DEX treatment of SAP‐ALI.

## 2 MATERIALS AND METHODS

### 2.1 Materials

Emodin (E8390, purity ≥ 98%), carboxymethylcellulose sodium (C8621) (Solarbio Science and Technology Co.), sodium taurocholate (86339) (Sigma‐Aldrich), dexamethasone (DEX) sodium phosphate injectable (Zhengzhou Ling Rui Pharmaceutical Co., Ltd), 10% neutral‐buffered formalin (Wexis Biotechnology Limited Company) and pentobarbital sodium (Merck KGaA) were the chemicals used in this study.

### 2.2 Animals

Sixty healthy male Sprague Dawley (SD) rats (specific pathogen‐free, eight weeks old, 180‐220 g) with animal licence number SYXK (Liao) 2013‐0003 were purchased from the Laboratory Animal Center of Dalian Medical University. Another animal licence number, SCXK (Liao) 2013‐0006 (with qualified number 211003700), was also acquired. The animals were housed in PVC cages. The temperature was controlled at 22 ± 2°C in a room with 12 hours light‐dark cycles and free access to standard laboratory food and drinking water for one week prior to the experiments. All animal experiments were conducted in accordance with the European Union animal management practices recommendations (1986). The protocols for model preparation and treatment were reviewed and approved by the Institutional Animal Care and Use Committee of Dalian Medical University (NO. AEE18019).

### 2.3 SAP model and experimental design

**Figure 1:**
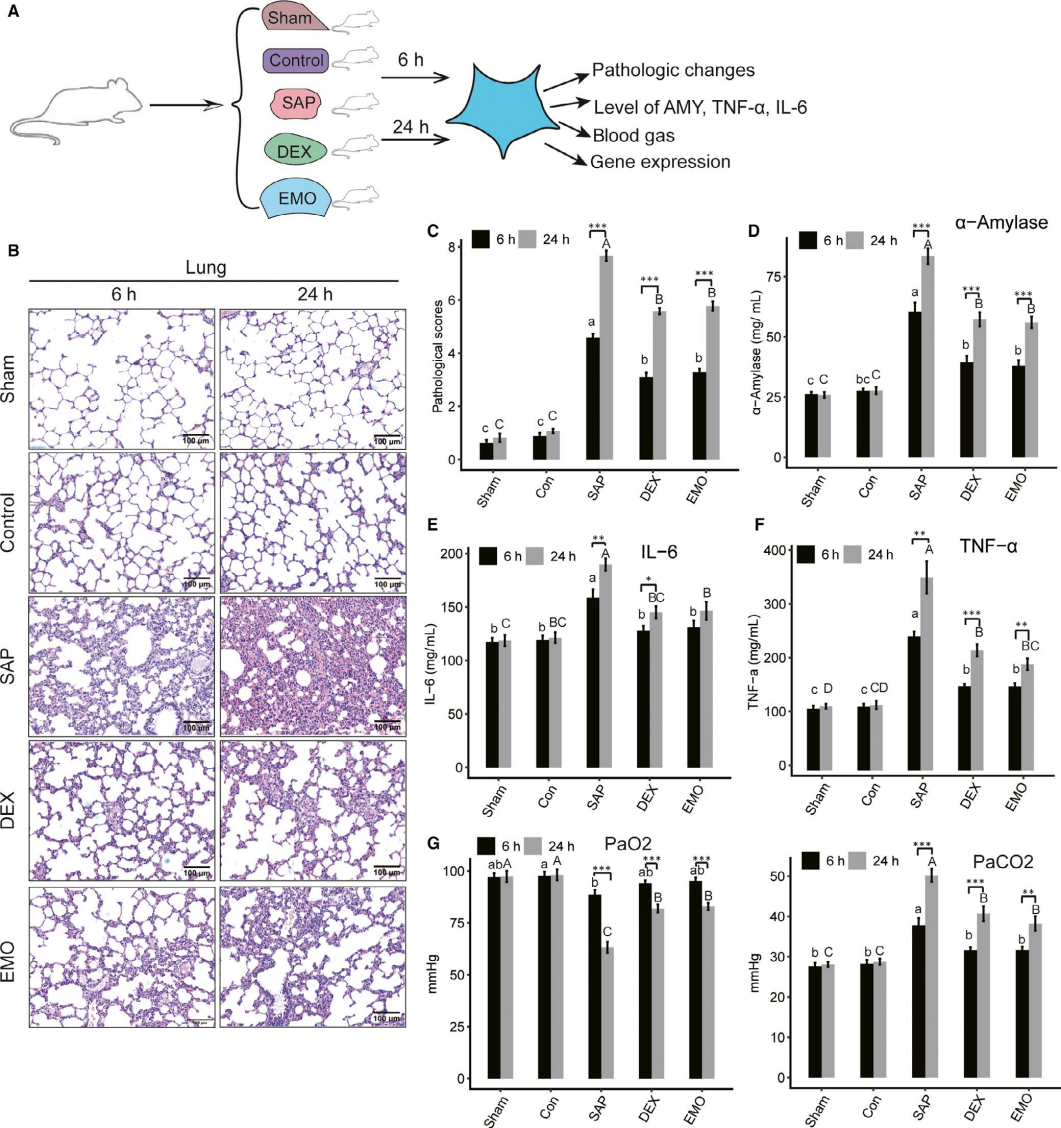
Pathologic characteristics of lung tissue from SAP‐ALI rats with drug treatment. A, The experimental design of this study. B, Haematoxylin‐eosin staining of lung tissue. A representative image of haematoxylin‐eosin staining of lung tissue was selected for each group. The scale bar is 100 μm. C, The analysis of pathological scores. D, Quantitative analysis of amylase (AMY). E, Quantitative analysis of TNF‐α. F, Quantitative analysis of IL‐6. G, Analysis of blood gas. PaO2 (left) and PaCO2 (right) in blood was measured. Data are presented as the means ± standard deviation (SD). Student's *t* test was performed to compare 6 and 24 hours for each group with significance set at a *P*‐value of <.05. *P < .05, **P < .01. Different letters on each bar (lowercase for 6 h and uppercase for 24 h, respectively) indicate significant difference between two groups (Tukey HSD, *P* < .05)

Animals were randomized into five groups (n = 12 per group); the control group, the sham‐operated group, the SAP model group, the SAP‐DEX‐treated group, and the SAP‐emodin‐treated group. Furthermore, each group was subdivided into 6 and 24 hours subgroups. Briefly, SD rats were denied food for 12 hours but had free access to water and were then given 1% pentobarbital sodium anaesthesia (intraperitoneal injection, 40 mg/100 g body weight) prior to the surgical operation. The SAP model was induced as previously described^33^ by administering fresh 5.0% sodium taurocholate (0.1 mL/100 g body weight) into the biliopancreatic duct by standard retrograde infusion. This procedure induced SAP and eventually resulted in a SAP‐induced ALI, which served as the model. Sterile saline of the same volume was given to the control group of animals. The sham group of animals had surgery to marginally rotate their pancreas. DEX (10 mg/kg body weight) was intravenously administered to the DEX group of animals at 2 and 12 hours post‐operation, and an equivalent volume of sterile saline was intravenously administered to the other groups of animals. Emodin (40 mg/kg body weight) was intragastrically given to the SAP‐emodin group at 2 and 12 hours post‐operation. An equal volume containing 0.5% carboxymethylcellulose sodium was intragastrically given to the other groups of animals. At 6 and 24 hours post‐operation, blood samples were taken via the abdominal aorta for arterial blood gas analysis and serum harvesting, and then stored at −80°C until used. At each time point, six rats were assessed. A portion of the lung tissue was fixed in 10% neutral phosphate formaldehyde. A needle was inserted from the ventriculus dexter into the pulmonary artery and irrigated with 9% sterile saline. The remaining portion of the lung was harvested and immediately stored at −80°C. Lung tissues from three of the six rats were used for RNA sequencing (RNA‐seq) and polymerase chain reaction (PCR) assessment. Figure 1A shows a brief flow diagram of the experimental design.

### 2.4 Histologic examination

Tissues were extracted from the pancreas and lungs and fixed in 10% neutral‐buffered formalin. The tissues were sectioned (size of 5 μm) and stained with haematoxylin and eosin (HE) as reported previously. Pathology tissue slices were critically examined with an optical microscope (Olympus BX53) by a pathologist blinded to the group and sample identities. Sections of the pancreas were scored for acinar necrosis, inflammation, haemorrhage and oedema based on a scale of 0‐4 for each parameter as previously described.^37^ Pulmonary histological assessment was based on oedema, leucocyte infiltration and haemorrhage on a scale of 0‐3 for each parameter.^38^

### 2.5 Measurement of AMY (α‐amylase), TNF‐α and IL‐6

Blood samples were collected from the abdominal aorta of the rats. After a 10 minutes centrifugation ( 845g), serum was obtained. AMY, TNF‐α and IL‐6 levels were determined with commercially available ELISA kits (Lengton Bioscience Co., LTD) in accordance with the manufacturer's protocols.

### 2.6 Analysis of blood gas

Blood taken from rat abdominal aortas was analysed with an automated analyzer for blood gas: RapidPoint 500 (SIEMENS, Berlin & Munich) at the Dalian Municipal Central Hospital Affiliated of Dalian Medical University.

### 2.7 RNA extraction, library construction and sequencing

Total RNA of each lung tissue sample was extracted using TRIzol Reagent (Ambion) according to the manufacturer's instructions. To eliminate any DNA present, RQ1 DNase (Promega) was added to the extracted total RNA. The absorbance of the purified RNA was measured to determine the quality and concentration with a Smartspec plus (BioRad) at 260 nm/280 nm (A260/A280). Agarose gel electrophoresis (1.5%) was conducted to verify the integrity of the RNA. For RNA‐seq library preparation, 10 μg of purified total RNA of each sample was purified and concentrated with oligo (dT)‐conjugated magnetic beads (Invitrogen). Polyadenylated mRNAs were used for the construction of a directional RNA‐seq library. Ion fragmentation of purified mRNAs was conducted at 95°C following end repair and 5′ adaptor ligation. The fragmented mRNAs were reverse‐transcribed with RT primer, known 3′ adaptors and random hexamers. Then, purification and amplification of the cDNAs were conducted. The 200‐500 bps of amplified cDNAs were collected. The cDNAs for each sample were quantified and stored at −80°C until sequencing. For high‐throughput sequencing, the libraries were prepared using the manufacturer's protocols and the Illumina HiSeq 2000 system for 150 nucleotide (nt) paired‐end sequencing (ABlife, Inc).

### 2.8 Raw data cleaning and mapping statistics

Raw reads with more than 2‐N bases were discarded. Reads were processed to clip the adaptor and remove the low‐quality bases (<20). Shorter reads (<16 nt) were discarded using the FASTX‐Toolkit (Version 0.0.13, http://hannonlab.cshl.edu/fastx_toolkit/). The rats' genome sequence and annotation file (rat_ensembl_v6) were downloaded from the Ensembl database (http://asia.ensembl.org/index.html). The resultant clean reads were mapped to the rat genome by TopHat2 allowing for two mismatches.^39^ Reads that mapped to multi genomic locations were discarded. Uniquely mapped reads were used to count mapped read numbers and calculate the fragments per kilobase per million (FPKM) value of each gene.

### 2.9 Prediction of lncRNA

The lncRNA pipeline prediction method was as described previously.^40^ Detailed pipeline prediction and filtering thresholds were described as follows: (a) based on the aligned RNA‐Seq results, transcripts were assembled using Cufflinks V2.2^41^ with default parameters. The assembled transcripts having FPKM of 0.1 or more were retained for further analysis. (b) The filtered transcripts were compared with known rat genes by Cuffcompare. Cufflinks novel transcripts containing intergenic and antisense regions were retained as the candidate lncRNAs. Transcripts that had 1000 base pairs (bp) overlapping with known coding genes were discarded. (c) Coding potential score (CPS) for transcripts was calculated with coding potential calculator (CPC) software.^42^ Transcripts that were candidate lncRNAs had CPS values below zero. (d) Transcripts with more than one exons and longer than 200 bases were defined as lncRNAs. (e) LncRNAs for each sample were combined by Cuffmerge embedded in Cufflinks as the final list of lncRNAs. After discarding the antisense reads, the FPKM value for each lncRNA gene was re‐calculated. (f) LncRNAs of human and mouse were downloaded from the GENCODE database.^43^ Each lncRNA was annotated by aligning the rat lncRNAs to lncRNAs of the human and mouse using Blastn software at E‐value < 1e‐3. In addition, rat lncRNAs sequences were aligned to NONCODE^44^ and LNCipedia^45^ databases using a similar procedure. The lncRNAs were named based on the best alignment score of the above databases. The naming rule for lncRNAs was used as described in a previous study.^46^

### 2.10 Differentially expressed genes and lncRNA (DEGs and DElncRNA)

Based on the expression level of each gene in each sample, DEGs and DElcnRNAs were identified by using edgeR as previously described.^47^ DEG and DElncRNA were defined as a 2‐fold change and a *P*‐value < .05. DEGs and DElncRNAs from different comparison groups were placed together and assigned to one union set for further analysis.

### 2.11 Weighted gene co‐expression network analysis

Weighted gene co‐expression network analysis (WGCNA) was performed to obtain an expression module that differentiated genes by their expression features.^48^ FPKM files of DEGs by any comparison were used as the input. Based on gene expression patterns, WGCNA resulted in several gene modules. Each gene module had an eigengene, which reflected the expression pattern of the module. WGCNA was also performed to produce a lncRNA module. For WGCNA, the soft threshold (power) for lncRNAs and mRNAs was set at 7 and 16, respectively, based on scale‐free topology criteria.

### 2.12 Co‐expression network analysis of mRNAs and lncRNAs

Correlation coefficients with a *P*‐value were calculated for each pair of mRNA‐lncRNA based on their expression level in each sample. Both the positive (correlation coefficient > 0) and negative (correlation coefficient < 0) pairs were considered. An absolute correlation coefficient > .7 and a *P*‐value < .01 were set as the threshold for co‐expression between two genes. The expression network was constructed with the filtered gene pair. Based on the expression pattern module by WGCNA and the lncRNA‐mRNA correlation pairs, a network of lncRNA‐mRNA pairs of each lncRNA or mRNA module was constructed. The proceeding analysis was lncRNA‐mRNA network dependent.

### 2.13 GO analysis

To predict gene function, Gene Ontology (GO) analysis was conducted using a KOBAS 2.0 server.^49^ The enrichment of genes in each pathway was determined by the hypergeometric test and Benjamini‐Hochberg FDR at the corrected *P*‐value < .05.

### 2.14 RT‐qPCR validation of DElncRNAs and DEGs

Quantitative real‐time PCR (RT‐qPCR) was performed to validate the RNA‐seq data. The expression of selected DEGs and DElncRNAs was quantified by RT‐qPCR using GAPDH as a reference. RT‐qPCR was conducted for RNA samples that were used for RNA‐seq. RT‐qPCR conditions were as follows: denaturing at 95°C for 10 minutes, 40 cycles of denaturing at 95°C for 15 seconds, with annealing and extension at 60°C for 1 minute. RT‐qPCR for each sample was replicated three times. Primers for RT‐qPCR are found in Table S1.

### 2.15 Statistical analysis

Unpaired two‐tailed *t* tests were performed to compare pathological evaluation and RT‐qPCR data from two different groups. Analysis of variance (ANOVA) with subsequent Tukey's honestly significant difference (HSD) test was performed to compare differences among groups for the pathological score, AMY, TNF‐α, IL‐6 and blood gas results. SPSS software Version 19 (IBM) was used for the statistical tests. Statistical significance was set at probability (*P*) values <.05 (*P* < .05). Each group had at least three biological replicates (n ≥ 3). The data are presented as means ± standard deviation (SD).

## 3 RESULTS

### 3.1 Both emodin and DEX protect rats from acute pancreatitis and acute lung injury

The effect of emodin on lung tissue gene expression profiles was explored by first evaluating the pathologic changes and key physiological indices of SAP‐ALI rats (Figure 1A). Significant pathologic changes were observed in the pancreatic and pulmonary tissues of rats in each group compared with the control and sham groups, suggesting that the SAP‐ALI rat model was established successfully (Figure 1B,C, and Figure S1A,B). The pathological lung injury was 100%, 66.7% and 50% for SAP, DEX and Emodin groups, respectively. In the pancreas of SAP‐6h and SAP‐24h rats, pancreatic oedema, a large number of inflammatory cell infiltrations, extensive haemorrhage and multiple pancreatic acinar necrotic cells were observed. These findings were more severe in the SAP‐24h rats. Similarly, severe pulmonary oedema, infiltration of a large number of inflammatory cells and haemorrhage in multiple areas were also observed in the lungs of the SAP‐6h and SAP‐24h rats. These findings were more severe in the SAP‐24h rats. Furthermore, pathological damage in the emodin and DEX groups was reduced significantly compared with the SAP group at both 6 and 24 hours (Figure 1B,C, and Figure S1A,B).

Compared to control and sham rats, AMY was elevated significantly in the SAP rats. The levels were higher in the SAP‐24h compared with the SAP‐6h rats. Emodin and DEX significantly decreased the level of AMY in SAP rats at both 6 and 24 hours (Figure 1D). Compared to control and sham rats, serum TNF‐α and IL‐6 levels were both dramatically increased in the SAP rats, with greater expression in the SAP‐24h rats compared with the SAP‐6h rats. Emodin and DEX significantly decreased the levels of TNF‐α and IL‐6 when compared to SAP rats at both 6 and 24 hours (Figure 1E,F).

Blood gas analysis showed reduced blood oxygen concentration and an elevated carbon dioxide level for the SAP rats, which was compensatory for SAP‐6h but not SAP‐24h rats. Emodin and DEX significantly improved breathing and blood gas indices compared with SAP groups, especially when the rats were in a decompensating stage (SAP‐24h group) (Figure 1G). These results demonstrate that both emodin and DEX ameliorate SAP‐induced ALI in rats.

### 3.2 Emodin up‐regulates the expression of mRNAs and lncRNAs in lung tissue of sham rats with SAP‐ALI while DEX produces an opposite effect

**Figure 2:**
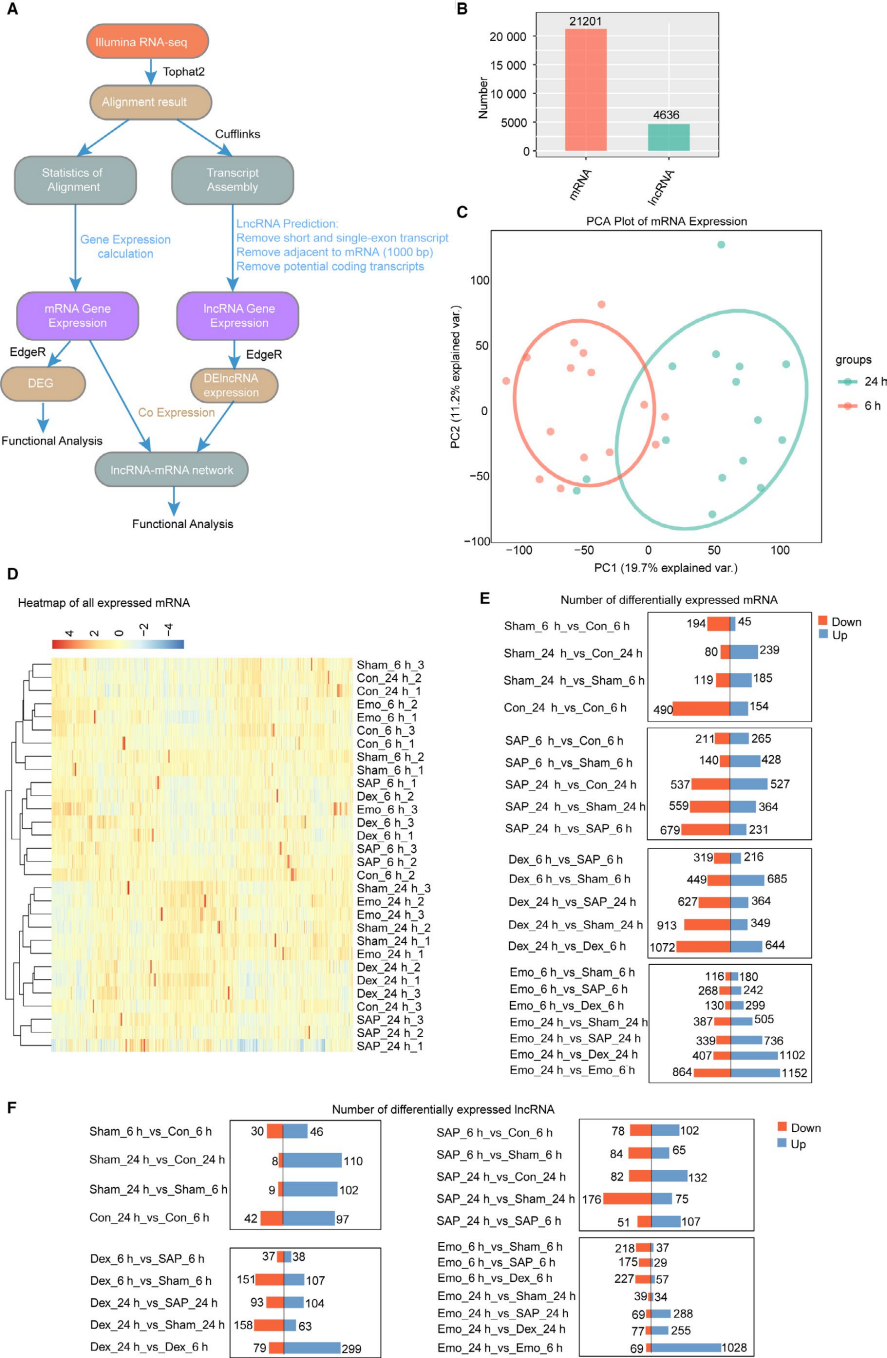
Effect of drug treatment on the gene expression profile of lung tissue from SAP‐ALI rats. A, The pipeline for identification and expression analysis of mRNA and LncRNA genes expressed in rat lung tissue. B, Numbers of expressed mRNA and lncRNA genes expressed in at least one sample (with FPKM > 0). C, Principal component analysis (PCA) of 30 distinct samples based on the expression level of mRNA genes. The samples were clustered by treatment time (6 and 24 h). D, Heat map of correlation coefficients of 30 samples according to expression level of mRNA genes. E, Distribution of the number of differentially expressed mRNAs between two groups. F, Distribution of the number of differentially expressed lncRNAs between two groups

To explore the transcriptome response to emodin, we performed RNA‐seq on the lung tissue of rats after SAP induction for 6 and 24 hours (SAP‐6h and SAP‐24h). SAP rats were treated with emodin (Emo‐6h and Emo‐24h) or DEX (DEX‐6h and DEX‐24h). We also assessed the lung tissue transcriptome of rats without SAP (Sham‐6h and Sham‐24h) and with SAP but injected with a salt solution into the pancreatic duct (Con‐6h and Con‐24h) (Figure 1A). In total, 30 transcriptome data sets were obtained (each sample had three replicates) providing an average of approximately 80 million PE‐end reads for analysis (Table S2). We then aligned clean reads to the reference sequence of the rat by TopHat2^39^ with two mismatches, and then detected and characterized the expression pattern of the annotated genes (Figure 2A and Table S3). In addition, Cufflinks was used to perform transcript assembly and transcript reconstruction. A total of 2930 novel lncRNA genes were identified after a filtering series (Figure 2A and Table S4). In total, there were 21 201 mRNA genes and 4636 lncRNA genes that were expressed in at least one sample (with FPKM > 0) (Figure 2B). These expressed mRNA and lncRNA genes were used to characterize changes in the transcriptome of the lung tissue of SAP rats after drug treatment.

Principal component analysis (PCA) was performed to explore the temporal expression patterns associated with all mRNAs and lncRNAs in the data sets. mRNA expression was separable between the 6 and 24 hours samples (Figure 2C). SAP‐24h and DEX‐24 were clustered together, but Emo‐24h was clustered with Sham‐24h, and Con‐24h (Figure 2C). Pearson correlations for all RNA‐seq sample pairs were performed, showing similar outcomes for mRNA expression (Figure 2D) and lncRNA expression (Figure S2A). LncRNA expression was obviously distinct between the 6 and 24 hours samples (Figure S2B). In particular, lung tissue mRNA expression with emodin treatment was more similar to the sham group than the DEX at 24 hours after SAP induction. These results demonstrate that the effect of SAP induction and drug treatment on gene expression in lung tissue is time‐dependent.

Furthermore, edgeR analysis was performed to identify differential expression of mRNAs (DEGs) and lncRNAs (DELnc) (≥two‐fold increase or decrease, FDR < 0.05) between the two groups at sample time points (6 or 24 hours) or between 6 and 24 hours for the same group. The results showed DEGs to be least for comparisons between sham group and con group at two‐time points, indicating similar mRNA expression. Notably, there were more up‐regulated DEGs than down‐regulated DEGs for Sham‐24h vs Sham‐6h, but this was reversed for Con‐24h vs Con‐6h and SAP‐24h vs SAP‐6h (Figure 2E). In fact, the salt solution injected into the pancreatic duct induced mild pancreatitis in rats, consistent with a similar trend in DEGs between 24 and 6 hours in the SAP and control groups. Thus, the sham group was selected as the actual control for comparison with SAP or drug treatment. As indicated above, more down‐regulated DEGs were detected in SAP vs Sham at similar time points. There were similar trends for DEGs in DEX vs SAP and DEX vs Sham at the same time points, as well as DEX‐24h vs DEX‐6h (Figure 2E). However, except for Emo‐6h vs SAP‐6h, there were more up‐regulated DEGs in comparisons involving Emo groups, which were similar to Sham‐24h vs Sham‐6h (Figure 2E). For DElncRNAs, there were similar trends for change in the direction and numbers (Figure 2F).

**Figure 3:**
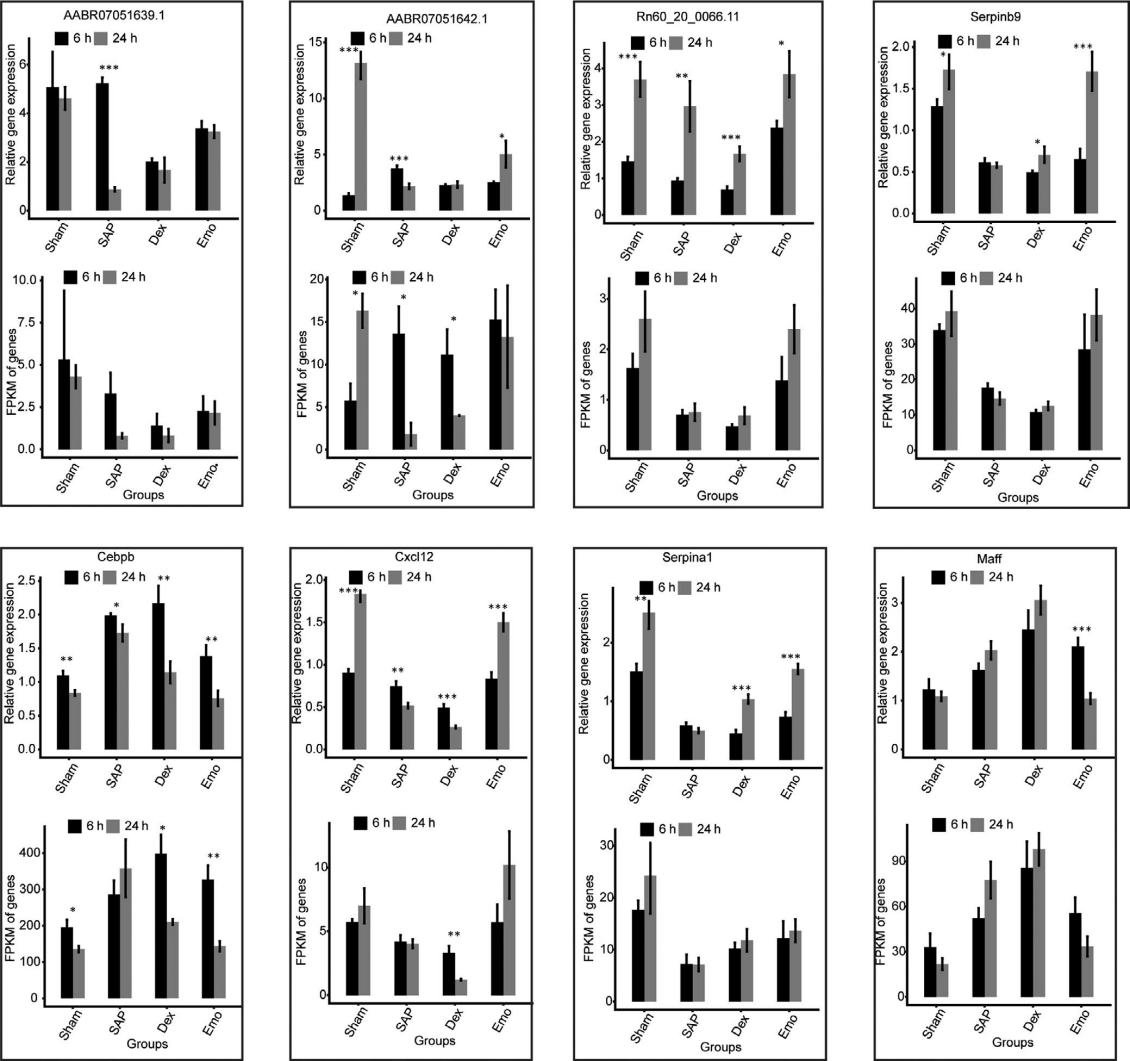
RT‐qPCR validation of DEGs and DELncRNAs. Relative expression levels of DEG and DELncRNAs by RT‐qPCR (up) and RNA‐seq (FPKM) (down). Data are presented as the mean ± standard deviation (SD). Student's *t* test was performed to compare 6 and 24 h with significance set at a *P*‐value of <.05. *P < .05, **P < .01

Expression of three lncRNA and ten mRNA was validated by qPCR (Figure 3 and Figure S3). These results indicated that emodin treatment differentially changes the expression of mRNA and LncRNA compared with the DEX treatment in lung tissues of SAP rats, which resulted in a sham‐like gene expression profiles after 24h.

### 3.3 The mRNA and lncRNA co‐expression modules associated with emodin treatment differed from those with DEX treatment

**Figure 4:**
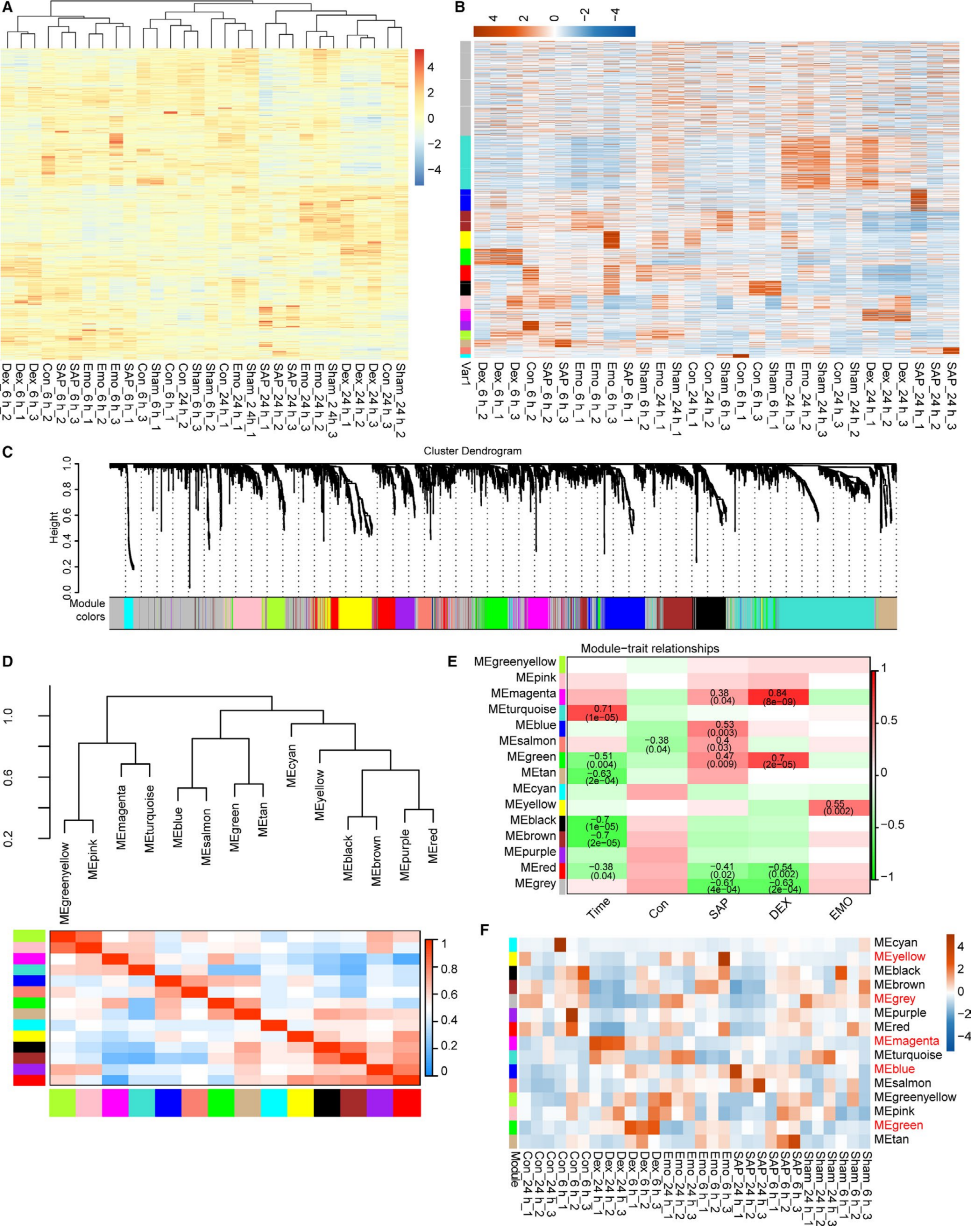
The co‐expression pattern of differentially expressed genes by weighted gene co‐expression correlation network analysis (WGCNA). A, Hierarchical clustering heat map of all samples based on all differentially expressed mRNA genes. B, Expression modules of differentially expressed mRNAs by WGCNA. C, Dendrogram of all differentially expressed mRNAs by hierarchical cluster analysis. Each co‐expressed gene was assigned to a module colour. D, Cluster analysis and heat map of each gene co‐expression module based on their correlations. E, Heat map of the correlations between each gene co‐expressions module (colour names) with traits (time, control, SAP, DEX and EMO). Pearson correlation coefficients with *P*‐values < .05 (in brackets) are presented. F, Eigengene pattern of each gene co‐expression module is presented by heat map

To characterize the expression patterns of lncRNA and mRNA, unsupervised hierarchical clustering analysis was performed for all unique DEmRNAs (4859) and DElncRNAs (1682). There were significant differences between the 6 and 24 hours samples, which indicated the time‐dependent expression of these DEmRNAs and DElncRNAs. Results showed a clear difference between SAP groups and DEX as well as Emo groups for DEmRNAs at 24 hours, although Emo was also different from the DEX group (Figure 4A). Similar to DEmRNA, DEX and Emo groups were completely separate from the SAP‐DEX groups for the DELcnRANs at 24 hours, except for Emo‐24h (Figure S4A). These results suggest that DEX and Emo treatments returned lung tissue mRNA and LncRNA expression levels of SAP‐induced rats to that of normal lung tissue of sham rats.

To further explore the expression pattern of DElncRNAs and DEmRNAs, WGCNA analysis was used to cluster all expression patterns. We identified 14 mRNA and 7 lncRNA co‐expression modules, respectively (Figure 4B and Figure S4B). DEmRNAs and DElncRNAs of each module had different expression patterns (Figure 4B and Figure S4B). Moreover, the DEmRNAs and DElncRNAs could be specifically clustered on the basis of the modules (Figure 4C and Figure S4C). In addition, all modules of DEmRNAs and DElncRNAs could further be clustered into eight and five groups, respectively, with gene expression showing similar patterns (Figure 4D and Figure S4D).

These lncRNA and mRNA modules could further be classified into temporal, SAP‐ temporal, DEX‐temporal and Emo‐temporal modules. For DEmRNA, six modules were correlated significantly with time (*P* < .05) (Figure 4E). Importantly, six modules were correlated with SAP induction (Figure 4E). As shown in Figure 4E, only one module (blue) inversely correlated with control (*P* < .05). There were four modules potentially positive or negative with DEX treatment with relatively high Pearson correlation coefficients, all of which correlated with SAP induction. In particular, one module (yellow) highly correlated with emodin treatment (Figure 4E). For the DElncRNA, three, one, one and two modules were significantly correlated with time, control, SAP and DEX, respectively (*P* < .05), but no module was significantly correlated with emodin treatment (Figure S4E). These results indicate that emodin treatment neutralizes SAP‐induced gene expression with little effect on basic gene expression. In particular, a heatmap of the eigengene expression of co‐expression modules showed similar expression for Emo and Sham‐Con groups (Figure 4F).

**Figure 5:**
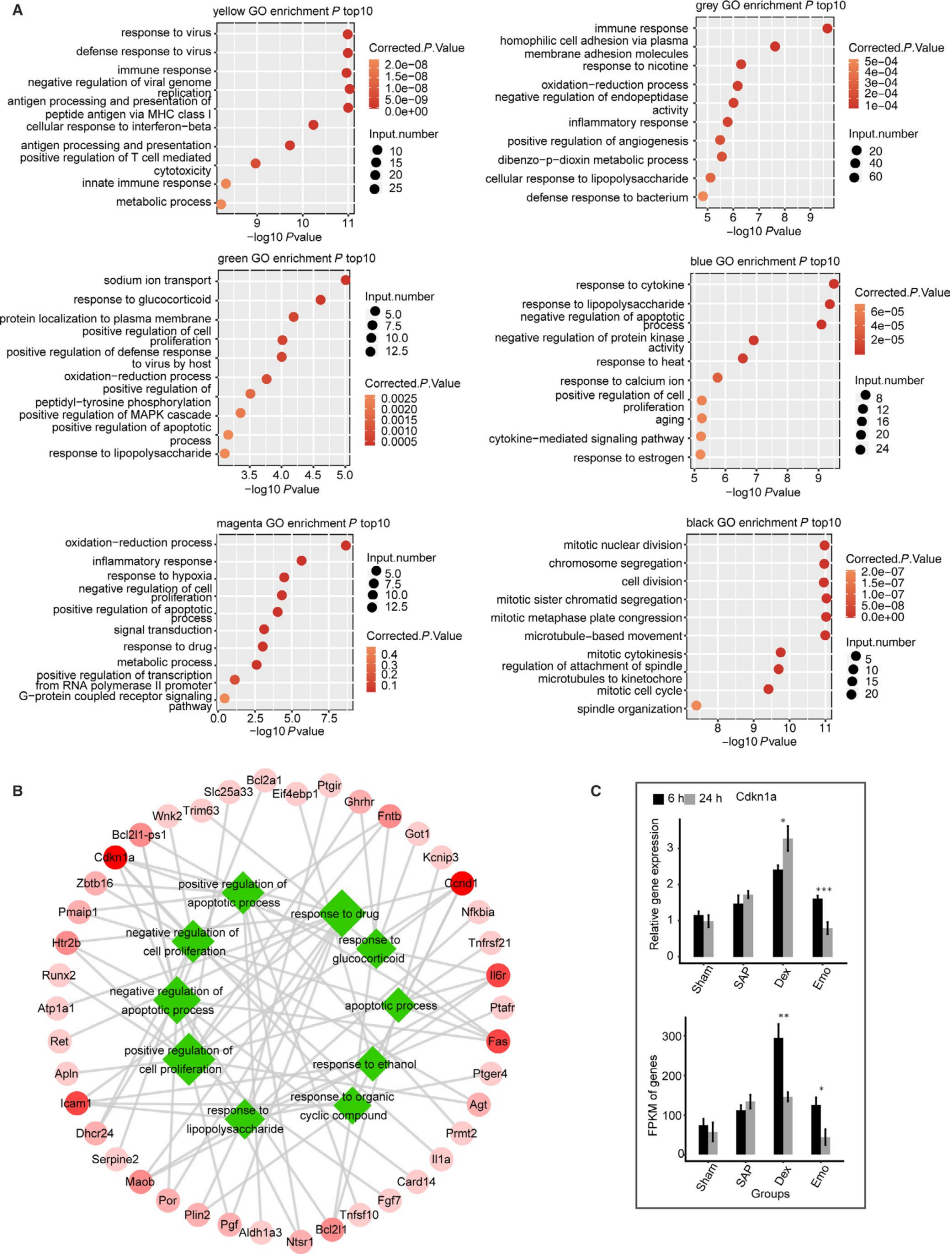
Functional analysis of mRNA genes from selected co‐expressed modules. A, The top10 GO analysis terms for mRNA genes from six co‐expressed modules. B, Functional analysis of mRNAs in green module. Genes enriched for two types of terms including response to chemicals and regulation of cell proliferation and apoptotic processes are presented. Green rhombuses are functional terms with size representing statistical significance. The red circles are the mRNAs. The line represents mRNAs involved terms with the degree of shade, the mRNA involvement and the number of terms involved. C, RT‐qPCR validation of the mRNA expression levels of Cdkn1a. RT‐qPCR (up) and RNA‐seq (FPKM) (down) are presented. Data are presented as means ± standard deviation (SD). Student's *t* test was performed to compare 6 and 24 h with significance set at a *P*‐value of <.05. *P < .05, **P < .01

GO analysis demonstrates that genes from the five different representative modules were significantly enriched in pathways associated with the stress response (Figure 5A), but not with the black module which was time‐dependent. The full list of the GO terms enriched in all six modules is found in Table S5. We focused on the green module which significantly correlated with time, SAP and DEX, but not Emo. Functional analysis of the interacted mRNAs in this module revealed that many genes were enriched in two‐term types including response to chemicals (glucocorticoid, lipopolysaccharide, drug, ethanol and organic cyclic compounds) and regulation of cell proliferation and apoptotic processes (Table S5). In addition, some genes were involved in several GO terms including response to chemicals and regulation of apoptosis (Figure 5B). mRNA expression levels of Cdkn1a were validated by qPCR, which showed the expression of this gene increased after SAP induction (Figure 5C). Importantly, for SAP induction, expression of Cdkn1a further increased in the DEX group, but not in the Emo group. These results indicate that with SAP induction, DEX, and Emo separately affect the expression level of genes for each mRNA and lncRNA co‐expression module in the lung tissue of rats.

### 3.4 Analysis of emodin‐associated lncRNA‐mRNA co‐expression networks

By exploring the function of lncRNA in the lung tissue of rats, a correlation matrix of all expressed 21 201 mRNA genes and 4636 lncRNAs was generated by computation of Pearson correlation coefficients for all paired combinations based on their expression in 30 transcriptomes. At a stringency of *P*‐value ≤ .01 and an absolute Pearson correlation coefficient of (PCC) ≥ .7, a total of 854 327 pairs were detected and co‐expressed between 4502 lncRNAs and 19 081 mRNAs (Table S6). Pairs that were positive were the predominant species, which was consistent with the co‐expression pattern of most study genes.^46^, ^50^ In particular, a relatively high percentage of lncRNA‐mRNAs (17.69%) and mRNA‐mRNAs (13.38%) were negative pairs, which was in contrast to the percentage of negative LncRNA‐LncRNA pairs (0.28%). These results indicate that a greater percentage of the lncRNAs involved were positive pairs, supporting the concept that lncRNAs promote expression of both mRNAs and lncRNAs in rats (Table S6).

**Figure 6:**
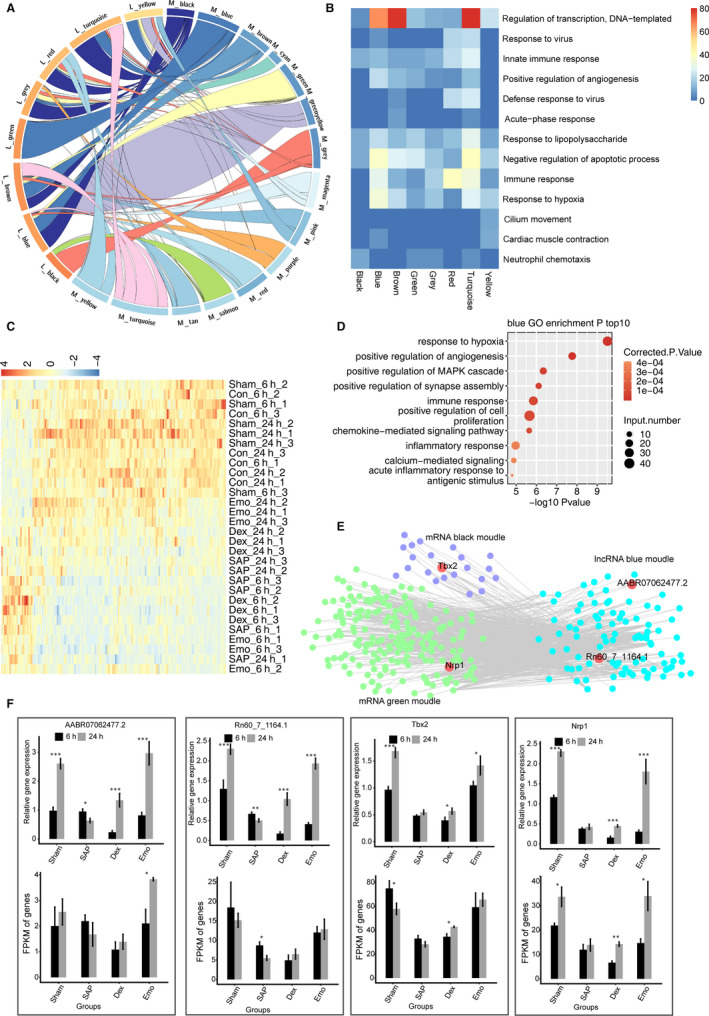
Analysis of lncRNAs‐mRNAs co‐expression networks. A, Interactions between each lncRNA module and each mRNA module are shown. B, GO analysis terms of mRNAs co‐expressed with each lncRNA module are presented by Heat map. The top GO analysis term for mRNAs of each lncRNA module is shown. Colour degree represents the normalization of statistical significance of each terms (−log 10 [*Q* value]). C, Heat map showing expression patterns of lncRNAs from the blue module. D, The top10 Go analysis terms for co‐expressed mRNAs with LncRNAs from the blue module. E, The co‐expression network of lncRNAs from the blue module and co‐expressed mRNAs from black and green modules. LncRNAs are on the right and co‐expressed mRNAs are on the left. F, RT‐qPCR validation of the mRNA expression levels of Tbx2, Nrp1 and LncRNAs AABR07062477.2 and Rn60_7_1164.1. RT‐qPCR (up) and RNA‐seq (FPKM) (down) are presented. Data are presented as means ± standard deviation (SD). Student's *t* test was performed to compare 6 and 24 h with significance set at a *P*‐value of <.05. *P < .05, **P < .01

To explore whether SAP or drug treatment affects lncRNA‐mRNA co‐expression, the number of LncRNA‐mRNA pairs between any two module pairs was determined (eight DElncRNAs and fifteen DEmRNA modules) (Table S7). We found that the number of module‐module co‐expression pairs was strong, which included exclusive pairs such as L_green‐M_blue, as well as multiple pairs such as L_blue with M_blue, M_green and M_grey (Figure 6A and Table S7). GO terms were further obtained for all mRNAs that interacted with each lncRNA module. Regulation of transcription, immune response and negative regulation of apoptotic processes were relatively enriched for mRNA genes co‐expressed with L_blue module lncRNAs. Immune response was mostly enriched for mRNA genes co‐expressed with L_red module lncRNAs (Figure 6B).

We further explored the function of L_blue lncRNA modules, whose expression levels were unregulated after emodin treatment (Figure 6C). GO analysis showed that mRNAs co‐expressed within this lncRNA module were enriched in apoptotic processes, positive regulation of apoptotic processes, response to a virus, regulation of transcription DNA‐template and positive regulation of cell migration (Figure 6D). We further generated co‐expression networks of lncRNA modules with their mRNA pairs and mapped their strength of interaction. The strength of interaction map revealed a strong correlation between mRNA genes and these lncRNA modules, including Tbx2 (Figure 6E). The expression levels of Tbx2, Nrp1 and lncRNAs (AABR07062477.2 and Rn60_7_1164.1) involved in the co‐expression network were subsequently validated by qPCR (Figure 6F). These genes showed higher expression levels in Sham and Emo groups than in SAP and DEX groups at 24 hours (Figure 6F). In particular, the co‐expression network of LncRNAs Rn60_7_1164.1 and AABR07062477.2 from the blue lncRNA module and Nrp1 from the green mRNA module was affected by emodin but not DEX. These results demonstrate the effect of emodin on the lncRNA‐mRNA co‐expression networks to be different from DEX.

## 4 DISCUSSION

ALI is the most severe complication resulting from SAP, responsible for 60%‐70% SAP‐associated deaths within the first week.^3^, ^4^ Many investigations have assessed the pathogenesis of SAP‐ALI and tested the effectiveness of potential drugs (eg. DEX and emodin) in animal models and SAP‐ALI patients.^6^ However, the molecular mechanisms involved in SAP‐ALI and effective drug treatment protocols have not been fully elucidated.^5^ In this study, we explored lncRNA‐mediated signal transduction networks underlying the pathogenesis and drug treatment responses in SAP‐ALI rats. This study showed that both emodin and DEX were therapeutic for SAP‐ALI, but the lung tissue gene expression profile with emodin treatment was similar to normal control rats. In particular, the lncRNA‐mRNA co‐expression modules affected by emodin were different from those of DEX, which indicates differences in molecular treatment effects for SAP‐ALI. Thus, this study provides evidence that emodin may be a suitable alternative or complementary medicine to DEX for the treatment of SAP‐ALI.

DEX is widely used to manage the inflammatory response during emergencies associated with various inflammatory diseases.^51^, ^52^ Emodin has also been reported having anti‐inflammatory, immunosuppressive, anti‐sepsis, anti‐fibrosis and anti‐neoplastic activities.^12^, ^53^, ^54^ In fact, both emodin and DEX ameliorate inflammatory responses and lung injury by decreasing serum TNF‐α and increasing the expression of AQP1 and AQP5 in the lungs of SAP‐ALI rats.^23^ Our previous study demonstrated that both emodin and DEX reduced lung injury by decreasing pre‐B‐cell colony‐enhancing factor expression and by the promotion of polymorphonuclear neutrophil apoptosis in a rat model.^22^ In this study, both DEX and emodin reduced lung injury in rats with SAP and decreased TNF‐α and IL‐6 expression. A previous study shows that DEX reduced the inflammatory response but failed to reduce the lung tissue injury in a SAP rat model.^10^ Moreover, like corticosteroids, high‐dose DEX produces frequent adverse effects including sleep insomnia, rapid weight gain, indigestion, glaucoma, hypokalaemia, pulmonary oedema, fungal infection and mood depression.^55^ We, therefore, speculated that the molecular mechanisms underlying the therapeutic effects of emodin and DEX on lung injury would be different in SAP‐ALI rats.

In this study, emodin and DEX differentially affected expression levels of protein‐encoding genes as well as lcnRNAs in the lung tissue of rats with SAP‐ALI. In particular, emodin predominately up‐regulated expression of mRNAs and lncRNAs in the lung tissue of rats with SAP‐ALI. A previous study showed that emodin attenuates apoptosis and inflammation induced by lipopolysaccharide via increases in the expression of the lncRNA, TUG1, in murine chondrogenic ATDC5 cells.^34^ DEX has been shown to induce cell death of human osteoblasts while ectopic overexpression of a lncRNA can inhibit DEX‐induced apoptosis and programmed necrosis in OB‐6 cells and primary human osteoblasts.^56^ Previous studies have shown that microRNAs, for example miR‐339‐3p, were aberrantly expressed during the pathogenesis of SAP‐ALI.^5^, ^30^ Emodin may inhibit inflammatory signalling pathways by increasing the expression of microRNA‐30a‐5p.^33^ DEX is known to regulate inflammation and immunity by decreasing microRNA‐155 in the liver of septic mice.^57^ These results indicate that emodin and DEX may adversely regulate expression of lncRNAs and microRNAs. MicroRNAs can inhibit the expression of mRNAs that have partial complementary sequences.^58^ Further, lncRNAs, for example LINCMD1, can regulate the abundance of miR‐133 and miR‐135 by binding and sequestering each, serving as microRNA sponges.^35^ It is possible to speculate that DEX and emodin affect the expression of lncRNAs that serve as sponges for microRNAs, affecting the expression of target mRNAs. Thus, DEX and emodin may have different effects on lncRNAs‐mRNA regulatory networks in the lung tissue of rats with SAP‐ALI. The role of microRNAs in such networks is an important area of investigation in future studies.

Weighted gene co‐expression network analysis (WGCNA) has been widely used to reveal the potential expression relationships between protein‐coding genes and lncRNAs.^48^ In order to deduce their potential function, lncRNAs need to be examined in relation to mRNAs, whose functions have already been annotated.^46^, ^59^, ^60^ As far as we know, no study has examined the expression profile of lncRNAs during SAP‐ALI with or without emodin and/or DEX treatment. In this study, WGCNA was used to identify DEX and emodin‐associated lncRNAs‐mRNAs co‐expression networks in the lung tissue of rats with or without SAP. Results showed that both DEX and emodin had specific associated lncRNA‐mRNA co‐expression modules. It is possible to speculate that co‐expressed mRNA‐lncRNA networks are involved in the mechanisms underlying the therapeutic or side effects of emodin and DEX treatment of rats with SAP‐ALI. For example, the blue lncRNA module and green mRNA module were significantly associated with DEX, but not with emodin. LncRNAs Rn60_7_1164.1 and AABR07062477.2 from the blue lncRNA module, respectively, showed a positive and negative relationship with expressions of neuropilin‐1 (Nrp1) from the green mRNA module. These results indicate that lncRNAs function by regulating the expression of potential target mRNA. Nrp1, a receptor for transforming growth factor β1, activates its latent form and enhances the activity of regulatory T cells^61^ involved in immune response regulation. Activation of the cellular immune response is essential to acute pancreatitis.^62^ The expression of Nrp1 is controlled by the TGF‐β signalling pathway, which can regulate the innate immune response negatively.^63^, ^64^ In particular, Nrp1 showed higher expression levels in Sham and Emo groups than in SAP and DEX groups at 24 hours. We, therefore, suggest that emodin but not DEX affects the lncRNA‐mRNA co‐expression module that promotes the immune response in lung tissue of rats with SAP‐ALI. Based on these results, future studies will be required to identify this potential lncRNA‐mRNA regulatory mechanism.

In conclusion, this study demonstrated both emodin and DEX to alleviate lung injury in rats with SAP‐ALI. However, emodin and DEX differentially affected the gene expression profile of lung tissue. Further, this study advanced the current understanding of possible treatment mechanisms by which emodin and DEX influence SAP‐ALI from the perspective of lncRNA‐mRNA co‐expression networks. Most importantly, this study showed that emodin‐associated lncRNA‐mRNA co‐expression networks were different from DEX networks (eg LncRNAs Rn60_7_1164.1‐Nrp1‐AABR07062477.2), suggesting different therapeutic mechanisms for SAP‐ALI. As such, this study provides preliminary evidence that emodin, as well as the Chinese herbal medicine rhubarb, may be an alternative or complementary medicine to the use of DEX for SAP‐ALI treatment. Moreover, this study provides data platforms and cues for future studies into the underlying mechanisms by which emodin and DEX differentially improve SAP‐ALI through signal transduction networks that involve lncRNAs.

## CONFLICT OF INTEREST

All authors declare no conflicts of interest.

## AUTHOR CONTRIBUTION

**Caiming Xu:** Conceptualization (lead); Formal analysis (lead); Methodology (lead); Writing‐original draft (lead). **Yalan Luo:** Methodology (equal); Writing‐original draft (supporting). **Michael Ntime:** Writing‐review & editing (equal). **Weili Quan:** Formal analysis (supporting); Methodology (supporting); Writing‐original draft (supporting). **Zhaoxia Li:** Data curation (equal); Methodology (supporting). **Qiushi Xu:** Methodology (supporting). **Liu Jiang:** Data curation (equal); Methodology (supporting). **Jingwen Zhang:** Funding acquisition (supporting). **Dong Shang:** Supervision (equal); Writing‐review & editing (equal). **Lei Li:** Formal analysis (equal); Methodology (supporting); Writing‐original draft (equal). **Guixin Zhang:** Conceptualization (supporting); Funding acquisition (equal); Supervision (equal); Writing‐review & editing (equal). **Hailong Chen:** Conceptualization (equal); Funding acquisition (lead); Project administration (lead); Writing‐review & editing (equal).

## COMPLIANCE WITH ETHICS REQUIREMENTS

This programme has compliance with ethics requirements.

## Supporting information

Table S3

Table S4

Table S5

Supplementary Material

## Data Availability

All data generated and analysed during this study have been included in this published article and its supporting information files. The data sets supporting the results of this article are available in the NCBI Gene Expression Omnibus and are accessible through GEO series accession number GSE151572. All other data are available upon reasonable request from the corresponding author.
