# Supplementary material for: Effect of emodin on long non‐coding RNA‐mRNA networks in rats with severe acute pancreatitis‐induced acute lung injury
Source: J Cell Mol Med. 2021 Jan 12;25(4):1851–66. doi: 10.1111/jcmm.15525 (PMC7882958; doi:10.1111/jcmm.15525)
Supplement: Supplementary file 4 — Supplementary Material [file JCMM-25-1851-s004.docx]

**Effect of emodin on long non-coding RNA-mRNA networks in rats with severe acute pancreatitis-induced acute lung injury**

Caiming Xu^a^, Yalan Luo^a^, Michael Ntim^b^, Weili Quan^c^, Zhaoxia Li^a^, Qiushi Xu^a^, Liu Jiang^a^, Jingwen Zhang^d^, Dong Shang^a^, Lei Li^e,^*****, Guixin Zhang^a,^*****, Hailong Chen^a,^*****

1. Department of General Surgery, The First Affiliated Hospital of Dalian Medical University, Dalian 116011, P.R. China; Institute (College) of Integrative Medicine, Dalian Medical University, Dalian 116044, P.R. China
2. Department of Physiology, Dalian Medical University, Dalian 116044, P.R. China.
3. Center for Genome Analysis, ABLife Inc, Wuhan 430075, P.R. China
4. Endoscopy Center, The First Affiliated Hospital of Dalian Medical University, Dalian 116011, P.R. China
5. Department of Vascular Surgery, The Second Affiliated Hospital of Dalian Medical University, Dalian 116027, P.R. China

***Corresponding authors at:** Department of Vascular Surgery, The Second Affiliated Hospital of Dalian Medical University, Zhongshan Road 467, Dalian 116027, P.R. China (Lei Li) or Department of Surgery, The First Affiliated Hospital of Dalian Medical University, Zhongshan Road 222, Dalian 116011, P.R. China (Guixin Zhang or Hailong Chen)

E-mail: leili_dmu@163.com (Lei Li), [zhangguixin@dmu.edu.cn](mailto:zhangguixin@dmu.edu.cn) (Guixin. Zhang), [chenhailong@dmu.edu.cn](mailto:chenhailong@dmu.edu.cn) (Hailong Chen)

**Supplementary Figures**


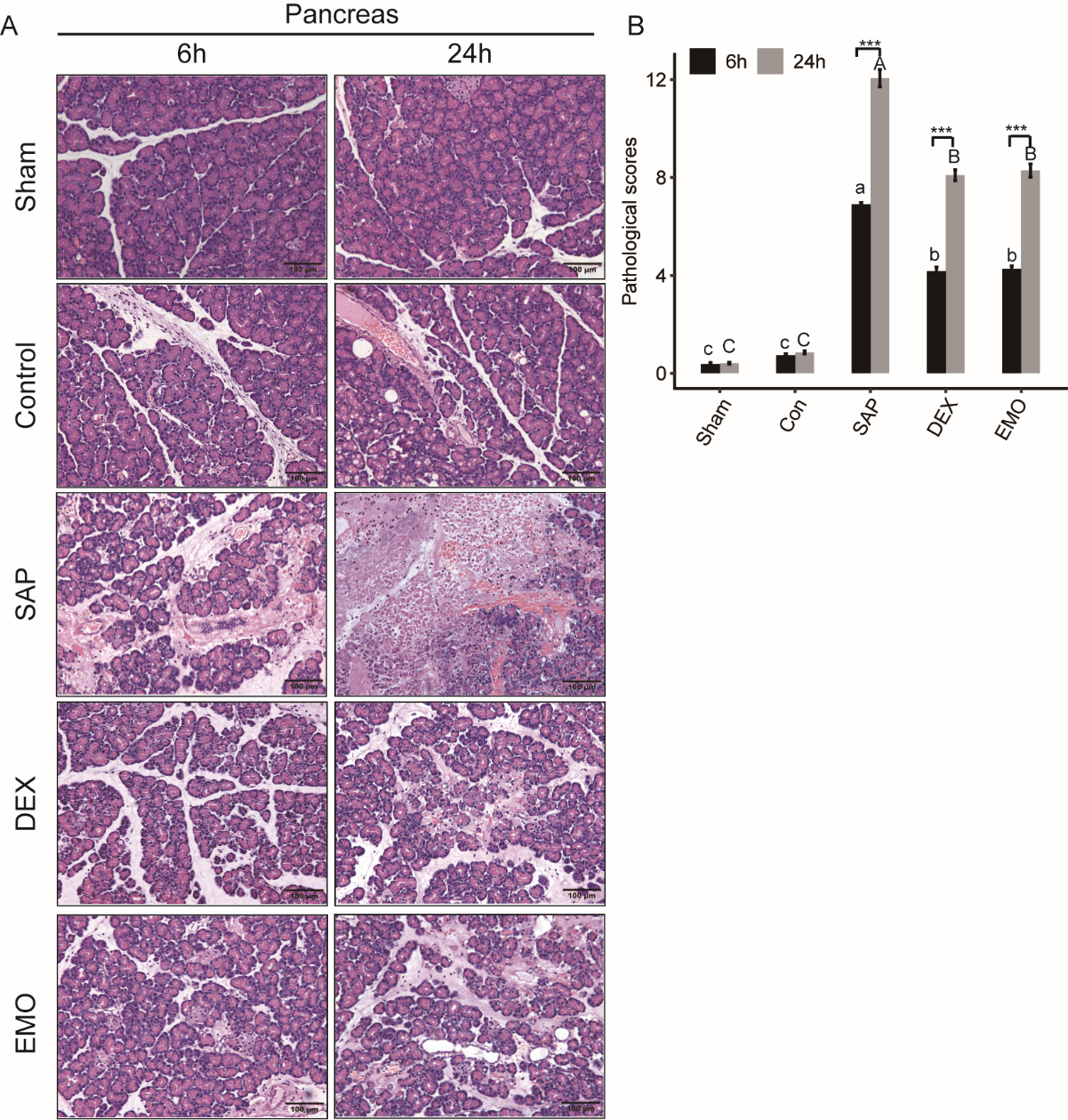


**Fig. S1 Pathologic characteristics of pancreatic tissues from SAP-ALI rats with drug treatments**. **A**. Hematoxylin-Eosin staining of pancreatic tissue. Representative images of Hematoxylin-Eosin staining of pancreatic tissue were selected from each group. The scale bar is 100 μm. **B**. Quantitative analysis of pathological scores. Data are presented as the mean ± standard deviation (SD). Student’s *t*-test was performed to compare 6h and 24h in each group with significance set at a *P* value of less than 0.05. * *P* < 0.05, ** *P* < 0.01. Different letters on the bar (lowercase for 6h and uppercase for 24h, respectively) indicate a significant difference between two groups (Tukey HSD, *P* < 0.05).


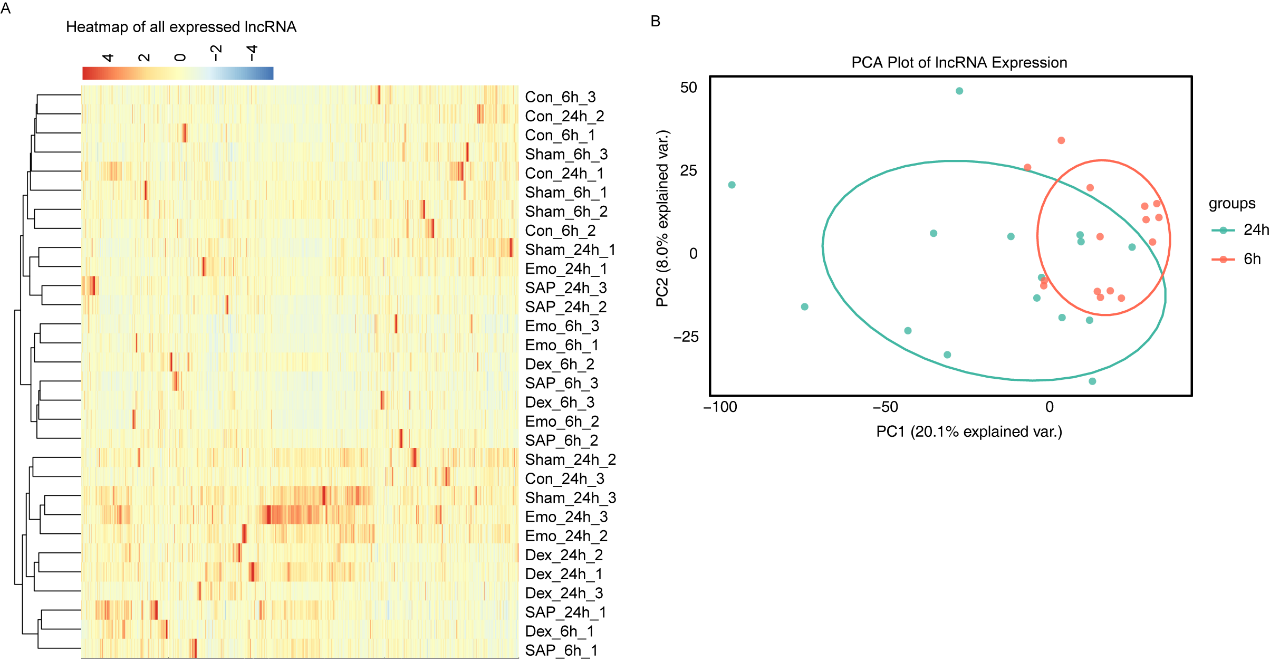


**Fig. S2 Effect of drug treatment on the LncRNA expression profile of lung tissue from SAP-ALI rats**. **A**. Heat map of the correlation coefficient for 30 samples based on the LncRNAs expression level. **B**. Principal component analysis (PCA) of 30 distinct samples from five treatment groups based on normalized LncRNAs expression level. The samples were grouped by treatment time (6h and 24h).


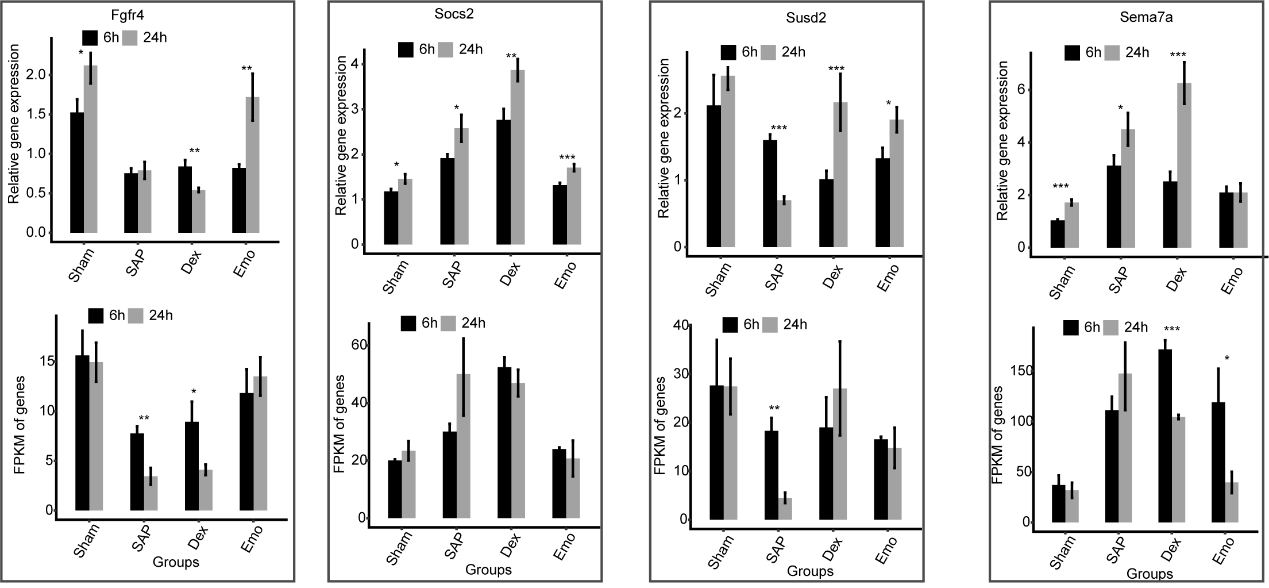


**Fig. S3 RT-qPCR validation of selected DEGs.** Data are represented as the mean ±standard deviation (SD). Student’s *t*-test was performed to compare 6h and 24h with significance set at a *P* value of less than 0.05. * *P* < 0.05, ** *P* < 0.01.


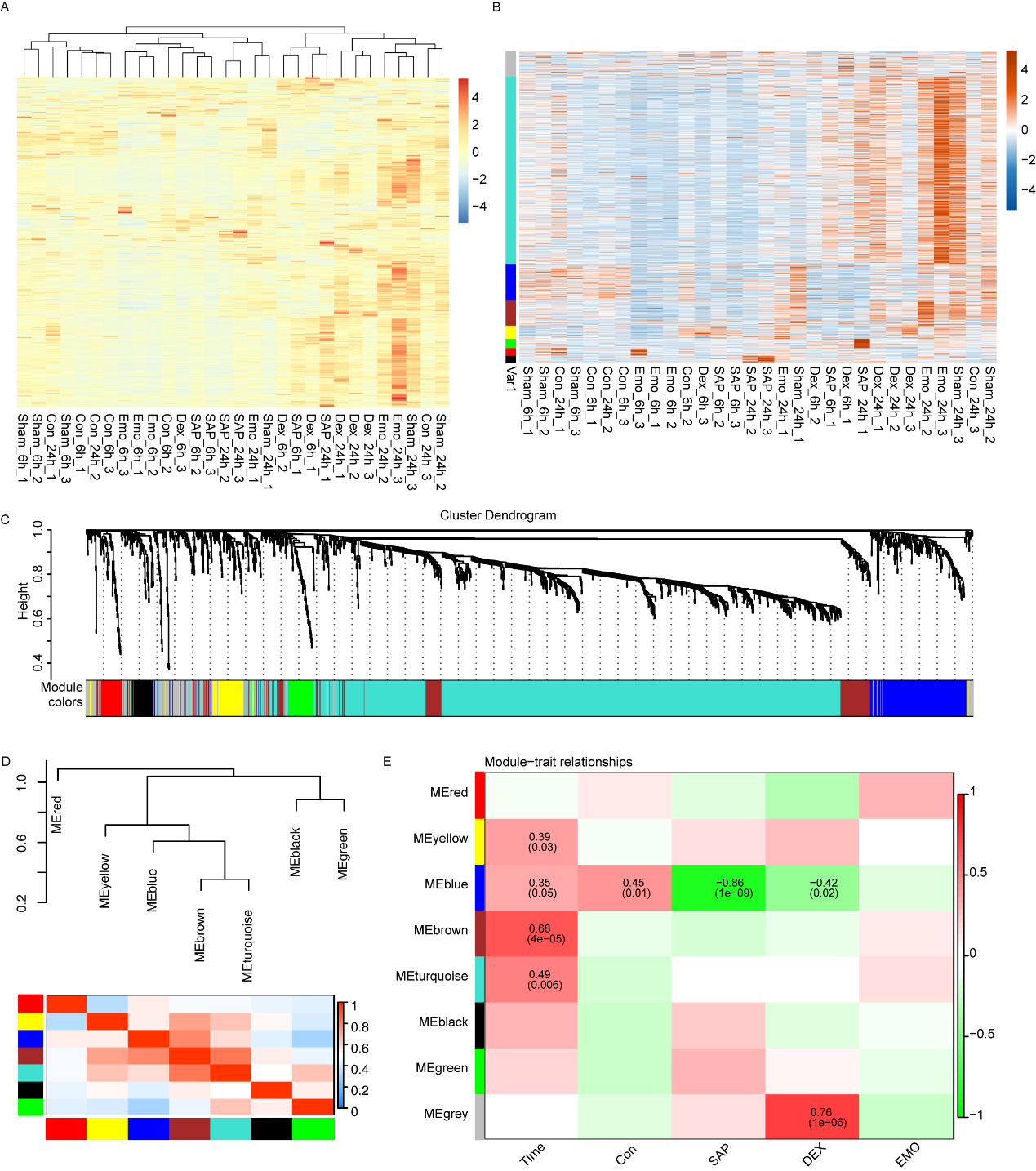


**Fig. S4 The co-expression pattern of differential expression of LncRNAs by weighted gene co-expression correlation network analysis (WGCNA)**. **A**. Hierarchical clustering and heat map of all samples based on all the differentially expressed LncRNAs. **B**. Expression modules of differentially expressed LncRNAs are presented by WGCNA. **C**. Dendrogram of all differentially expressed LncRNA by hierarchical cluster analysis. Each co-expressed gene was assigned with module colours. **D**. Cluster analysis and heat map of each gene co-expression modules based on their correlations. **E**. Heat map of the correlations between each gene co-expression module (colour names) with traits (time, control, SAP, DEX, and EMO). Pearson correlation coefficients with and P values < 0.05 (in brackets) are presented.

**Supplementary Tables**

**Table S1. Primers that were used for qPCR in DEGs and DELncRNAs**

| **Gene name** | **Forward primers** | **Reverse primers** |
| --- | --- | --- |
| **DEGs** |  |  |
| Tbx2 | AGCTAACGCTGCCCACTCT | CAGGACGAGGCATCGGATTC |
| Serpinb9 | CAGAGGAGATGCTCACAGGA | TCTGAAGCAAATGGCACCTTT |
| Nrp1 | TGGTGAGCCCTGTGGTCTA | TTCCTTCCAGTGGTCTCCTTG |
| Cxcl12 | CAGTCAGCCTGAGCTACAGAT | TGAAGGGCACAGTTTGGAGT |
| Fgfr4 | CTCTCGCAGCAGTGAGAACAG | TGGTCCTTCGGGATCCTGCT |
| Susd2 | GCGACCACTTCTGCGTTCT | AGGCTCTTCAGGCGATGCT |
| Cebpb | AGCGACGAGTACAAGATGC | GCTGCTCCACCTTCTTCTG |
| Sema7a | CAACCCATACCCACGGAAAC | GAGTGGAACAATGGTGTCTTCA |
| Cdkn1a | GTGATGTCCGACCTGTTCCA | CGTCTCAGTGGCGAAGTCA |
| Maff | GCGTCATCACCATTGTCAAGT | CAGAAGGCACTCAGGCTCTT |
| Socs2 | TGGCAATAGGTTAGGACTCACA | ACAAGAATGTCACGCTACACAG |
| Serpina1 | CGTTGCCCAGGTAATCCATC | CCATTCAATCCTGAGCACACTA |
| Rat-GAPDH | AAGTTCAACGGCACAGTCAAG | ACATACTCAGCACCAGCATCA |
|  |  |  |
| **LncRNAs** |  |  |
| Rn60_7_1164.1 | TGCTTCCAGTGCTTCAAGG | GTGTAAAGAAGAACCTCATTGC |
| AABR07051642.1 | TCCAGATGACACAGTCTCCAG | CTTCTGCTGATACCACGCTAA |
| Rn60_20_0066.11 | GCAGGTTCAGAGGTGTAGCA | GGAGCAGCGCAGAAGAGAA |
| AABR07062477.2 | TGGTGTCCGACATCTGTTGG | GCGGCAGAACCTTGAGCAT |
| AABR07051639.1 | CGAATGTCTAGCAAGTGAGGA | AATAAGTCGCAGCATCTTCAGA |

**Table S2. Summary of sample names, description, the RNA-seq sequencing information and mapping results in each sample**

| SampleID | raw_total | clean_total | ratio_total | raw_base | clean_base | ratio_base | uniqtag | Q20 | Q30 | GC | DUP |
| --- | --- | --- | --- | --- | --- | --- | --- | --- | --- | --- | --- |
| Con_6h_1 | 80275308 | 74669192 | 93.02% | 12.041G | 10.763G | 89.39% | 36399718(48.75%) | 97.54 | 92.91 | 53% | 87.48% |
| Con_6h_2 | 73310736 | 68094923 | 92.89% | 10.997G | 9.814G | 89.25% | 36397207(53.45%) | 97.65 | 93.15 | 51% | 85.41% |
| Con_6h_3 | 72702974 | 67727897 | 93.16% | 10.905G | 9.790G | 89.77% | 35419545(52.30%) | 95.22 | 87.77 | 52% | 85.86% |
| Sham_6h_1 | 85510686 | 79476479 | 92.94% | 12.827G | 11.487G | 89.55% | 41295241(51.96%) | 95.26 | 87.96 | 50% | 85.85% |
| Sham_6h_2 | 74486042 | 69319361 | 93.06% | 11.173G | 10.020G | 89.68% | 36549350(52.73%) | 96.64 | 90.81 | 50% | 84.91% |
| Sham_6h_3 | 72748986 | 67252196 | 92.44% | 10.912G | 9.709G | 88.97% | 34536540(51.35%) | 95.14 | 87.65 | 52% | 85.87% |
| Con_24h_1 | 66961038 | 62710166 | 93.65% | 10.044G | 8.963G | 89.24% | 32937290(52.52%) | 97.67 | 93.26 | 51% | 86.35% |
| Con_24h_2 | 78769430 | 73236821 | 92.98% | 11.815G | 10.568G | 89.44% | 37818500(51.64%) | 95.29 | 87.98 | 51% | 86.34% |
| Con_24h_3 | 67965224 | 62766850 | 92.35% | 10.195G | 9.055G | 88.82% | 34350676(54.73%) | 97.68 | 93.2 | 51% | 84.28% |
| Sham_24h_1 | 89693622 | 82148536 | 91.59% | 13.454G | 11.788G | 87.62% | 43296742(52.71%) | 96.19 | 89.76 | 50% | 86.02% |
| Sham_24h_2 | 83662548 | 77283360 | 92.38% | 12.549G | 11.084G | 88.33% | 41893073(54.21%) | 97.67 | 93.19 | 49% | 84.10% |
| Sham_24h_3 | 89002786 | 82590335 | 92.80% | 13.350G | 11.856G | 88.81% | 44028662(53.31%) | 95.37 | 88.24 | 50% | 83.24% |
| SAP_6h_1 | 76832424 | 71901241 | 93.58% | 11.525G | 10.351G | 89.82% | 28385659(39.48%) | 97.68 | 93.54 | 51% | 85.60% |
| SAP_6h_2 | 96533192 | 92307221 | 95.62% | 14.480G | 13.294G | 91.81% | 34955489(37.87%) | 97.86 | 93.85 | 50% | 87.27% |
| SAP_6h_3 | 87251468 | 84218549 | 96.52% | 13.088G | 12.122G | 92.62% | 32147909(38.17%) | 97.6 | 93.32 | 51% | 87.32% |
| Dex_6h_1 | 85383856 | 82884269 | 97.07% | 12.808G | 11.949G | 93.29% | 30943242(37.33%) | 97.76 | 93.75 | 51% | 87.29% |
| Dex_6h_2 | 78442186 | 76302503 | 97.27% | 11.766G | 10.995G | 93.44% | 28749764(37.68%) | 97.82 | 93.86 | 51% | 86.85% |
| Dex_6h_3 | 87169134 | 84956092 | 97.46% | 13.075G | 12.247G | 93.66% | 31007380(36.50%) | 97.88 | 94.01 | 52% | 88.12% |
| Emo_6h_1 | 74055258 | 72097609 | 97.36% | 11.108G | 10.398G | 93.60% | 26990342(37.44%) | 97.86 | 93.95 | 52% | 87.47% |
| Emo_6h_2 | 79639446 | 77388725 | 97.17% | 11.946G | 11.136G | 93.22% | 29282635(37.84%) | 97.8 | 93.79 | 51% | 87.61% |
| Emo_6h_3 | 75846052 | 73834584 | 97.35% | 11.377G | 10.637G | 93.49% | 27973653(37.89%) | 97.83 | 93.87 | 52% | 87.63% |
| SAP_24h_1 | 81524814 | 78957586 | 96.85% | 12.229G | 11.329G | 92.64% | 29549205(37.42%) | 97.67 | 93.58 | 50% | 87.04% |
| SAP_24h_2 | 86338710 | 83772704 | 97.03% | 12.951G | 12.027G | 92.87% | 30773984(36.74%) | 97.56 | 93.29 | 51% | 87.89% |
| SAP_24h_3 | 79091640 | 76902991 | 97.23% | 11.864G | 11.043G | 93.08% | 29483739(38.34%) | 97.64 | 93.49 | 50% | 86.74% |
| Dex_24h_1 | 96612792 | 93568404 | 96.85% | 14.492G | 13.421G | 92.61% | 37625935(40.21%) | 97.48 | 93.13 | 49% | 85.15% |
| Dex_24h_2 | 77810472 | 75721987 | 97.32% | 11.672G | 10.886G | 93.27% | 29784678(39.33%) | 97.71 | 93.68 | 50% | 86.32% |
| Dex_24h_3 | 78694320 | 75651060 | 96.13% | 11.804G | 10.846G | 91.88% | 30461782(40.27%) | 97.32 | 92.72 | 50% | 86.23% |
| Emo_24h_1 | 71934308 | 67736060 | 94.16% | 10.790G | 9.728G | 90.16% | 28735183(42.42%) | 97.81 | 93.69 | 50% | 84.91% |
| Emo_24h_2 | 82128690 | 78628767 | 95.74% | 12.319G | 11.269G | 91.47% | 33805744(42.99%) | 97.16 | 92.36 | 49% | 84.16% |
| Emo_24h_3 | 74837172 | 72171050 | 96.44% | 11.226G | 10.381G | 92.47% | 31594472(43.78%) | 97.32 | 92.74 | 50% | 82.61% |

**Table S3. The expression level of mRNA genes (FPKM)**

This file is not suitable for merging into this word file for submission. It is available upon request from the corresponding author.

**Table S4. The expression level of all LncRNA genes (FPKM).**

This file is not suitable for merging into this word file for submission. It is available upon request from the corresponding author.

**Table S5 List of GO terms enriched by mRNA gene from selected co-expression modules**

This file is not suitable for merging into this word file for submission. It is available upon request from the corresponding author.

**Table S6. Correlation pairs statistics with a different threshold of lncRNA-mRNA, lncRNA-lncRNA, and mRNA-mRNA**

| **Network** | **Threshold** | **Pair number** | **Positive pair number(Per)** | **Negative pair number(Per)** | **LncRNA number in pairs** | **mRNA number in pairs** |
| --- | --- | --- | --- | --- | --- | --- |
| **LncRNA-mRNA** | abs(cor)>=0.5, Pvalue<=0.05 | 4146651 | 2823165 | 1323486 | 4636 | 21201 |
|  | abs(cor)>=0.7, Pvalue<=0.01 | 854327 | 703114 | 151213 | 4502 | 19081 |
| **LncRNA-LncRNA** | abs(cor)>=0.5, Pvalue<=0.05 | 759933 | 744348 | 15585 | 4636 | -- |
|  | abs(cor)>=0.7, Pvalue<=0.01 | 293831 | 292988 | 843 | 3650 | -- |
| **mRNA-mRNA** | abs(cor)>=0.5, Pvalue<=0.05 | 17246972 | 10990598 | 6256374 | -- | 21203 |
|  | abs(cor)>=0.7, Pvalue<=0.01 | 6178066 | 4535033 | 825150 | -- | 19524 |

**Table S7 Relationship between lncRNA and mRNA modules based on the correlation network**

|  |  | **LncRNA module** | | | | | | | |
| --- | --- | --- | --- | --- | --- | --- | --- | --- | --- |
|  |  | turquoise | blue | brown | grey | yellow | green | red | black |
| **mRNA module** | black | 146 | 43 | 9 | 32 | 1 |  | 27 | 0 |
|  | blue | 1437 | 844 | 173 | 24 | 10 | 3395 | 9 | 1 |
|  | brown | 425 | 147 | 61 | 11 | 2 | 7 | 0 | 0 |
|  | cyan | 3 | 1 | 0 | 14 | 0 | 0 | 0 | 0 |
|  | green | 84 | 893 | 201 | 131 | 12 | 56 | 19 | 1 |
|  | greenyellow | 33 | 13 | 3 | 4 | 178 | 0 | 0 | 0 |
|  | grey | 1234 | 1001 | 378 | 200 | 147 | 24 | 107 | 209 |
|  | magenta | 975 | 87 | 71 | 266 | 271 | 13 | 3 | 0 |
|  | pink | 57 | 3 | 62 | 5 | 1458 | 0 | 0 | 0 |
|  | purple | 72 | 45 | 1 | 624 | 29 | 0 | 3 | 0 |
|  | red | 24 | 170 | 112 | 73 | 2 | 1 | 483 | 0 |
|  | salmon | 83 | 1 | 19 | 24 | 0 | 71 | 0 | 445 |
|  | tan | 29 | 101 | 20 | 177 | 1 | 1 | 0 | 0 |
|  | turquoise | 100990 | 1200 | 17091 | 2 | 374 | 20 | 0 | 8 |
|  | yellow | 911 | 2 | 10 | 1 | 0 | 3 | 2621 | 0 |
